# A Nitric Oxide-Donating Statin Decreases Portal Pressure with a Better Toxicity Profile than Conventional Statins in Cirrhotic Rats

**DOI:** 10.1038/srep40461

**Published:** 2017-01-13

**Authors:** Sarai Rodríguez, Imma Raurell, Manuel Torres-Arauz, Teresa García-Lezana, Joan Genescà, María Martell

**Affiliations:** 1 Liver Diseases Laboratory, Liver Unit, Department of Internal Medicine, Hospital Universitari Vall d’Hebron, Institut de Recerca (VHIR), Universitat Autònoma de Barcelona, Barcelona, 08035, Spain; 2 Centro de Investigación Biomédica en Red de Enfermedades Hepáticas y Digestivas (CIBERehd), Instituto de Salud Carlos III, Madrid, 28029, Spain

## Abstract

Statins present many beneficial effects in chronic liver disease, but concerns about safety exist. We evaluated the hepatic effects of a nitric oxide-releasing atorvastatin (NCX 6560) compared to conventional statins. Simvastatin, atorvastatin and NCX 6560 were evaluated in four-week bile duct-ligated rats (BDL) simulating decompensated cirrhosis and in thirteen-week carbon tetrachloride (CCl_4_) intoxicated rats, a model of early cirrhosis. In the BDL model, simvastatin treated rats showed high mortality and the remaining animals presented muscular and hepatic toxicity. At equivalent doses, NCX 6560 eliminated hepatic toxicity and reduced muscular toxicity (60–74%) caused by atorvastatin in the more advanced BDL model; toxicity was minimal in the CCl_4_ model. Atorvastatin and NCX 6560 similarly reduced portal pressure without changing systemic hemodynamics in both models. Atorvastatin and NCX 6560 caused a mild decrease in liver fibrosis and inflammation and a significant increase in intrahepatic cyclic guanosine monophosphate. NCX 6560 induced a higher intrahepatic vasoprotective profile (activated endothelial nitric oxide synthase and decreased platelet/endothelial cell adhesion molecule-1), especially in the CCl_4_ model, suggesting a higher benefit in early cirrhosis. In conclusion, NCX 6560 improves the liver profile and portal hypertension of cirrhotic rats similarly to conventional statins, but with a much better safety profile.

Portal hypertension of cirrhosis is initiated by an increased hepatic resistance to portal blood flow and maintained and aggravated by an augmented portal inflow due to splanchnic arterial vasodilation and hyperdynamic syndrome. The increase in intrahepatic vascular resistance (IHVR) is mainly due to architectural distortion (fibrosis, nodules) and imbalance between vasoconstrictors and vasodilators leading to endothelial dysfunction and an increment in intrahepatic vascular tone^1^,^2^,^3^.

Many therapeutic strategies aimed at decreasing IHVR (e.g.: antioxidants, nitric oxide (NO) donors, statins) have been developed^3^,^4^,^5^. NO donors such as isosorbide-5-mononitrate have been shown to decrease portal pressure (PP)^6^, but their use has been limited due to systemic vasodilatory effects^7^,^8^. Statins have also been shown to ameliorate portal hypertension and liver function^9^,^10^,^11^,^12^. Although statin therapy is commonly regarded as well tolerated in patients with mild liver disease^13^,^14^,^15^, serious adverse effects have also been reported^16^,^17^,^18^.

Statins exert beneficial lipid-independent pleiotropic effects^19^ through 3-hydroxy-3-methylglutaryl-coenzyme A (HMG-CoA) reductase inhibition, reducing mevalonate production and therefore, the synthesis of isoprenoids, farnesyl pyrophosphate and geranylgeranyl pyrophosphate. These compounds, in turn, serve as lipophilic attachments for various signaling proteins, such as the small Ras, Rac, and Rho GTPases, which are involved in cell proliferation, cytoskeletal organization, and activation of transcription factors^19^,^20^,^21^.

Mechanisms involved in the beneficial intrahepatic effects of statins include improvement of endothelial dysfunction through increasing endothelial nitric oxide synthase (eNOS) activity (by an increment in eNOS phosphorylation at serine 1176 [1177 in the human sequence])^10^ and inhibition of RhoA/Rho-kinase^11^. Statins also cause attenuation of both liver angiotensin II-induced inflammatory actions^22^ and hepatic fibrosis via decreased turnover of hepatic stellate cells and downregulation of profibrotic cytokine expression^23^,^24^. Recently, an overexpression of the transcription factor Krüppel-like factor 2 (KLF2) that orchestrates a variety of vasoprotective pathways induced by statins has also shown to confer hepatic endothelial vasoprotection and stellate cells deactivation^25^,^26^.

Among statins, simvastatin has been tested in clinical trials and has shown to lower portal pressure and hepatic resistance^9^,^12^ and even improved survival after variceal bleeding in patients with cirrhosis^27^. In cirrhotic rats though, atorvastatin showed a greater PP lowering effect together with an attenuation of fibrosis and hepatic stellate cell activation^11^,^23^ and a reduction in neo-angiogenesis in the splanchnic circulation^28^. NCX 6560, a NO-releasing derivative of atorvastatin, exerts greater lipid-lowering, antithrombotic and anti-inflammatory effects than atorvastatin and prevents statin-induced myopathy^29^,^30^,^31^,^32^.

Thus, the aim of this study was to evaluate whether NCX 6560 is superior to conventional statins (simvastatin, atorvastatin) in reducing portal hypertension and intrahepatic vascular resistance, while decreasing the potential side effects of statins.

## Results

In the bile duct-ligation (BDL) model, cirrhosis, defined by macroscopic observations and histological findings, was present in all rats, 30% of the animals presented ascites and some animals died before completing statin treatment (see mortality rates in Table 1), whereas in the carbon tetrachloride (CCl_4_) cirrhotic model, all rats that followed the 13-week CCl_4_ inhalation protocol exhibited extensive fibrosis, but none presented ascites.

### Adverse events associated with statin treatment in cirrhotic rats

As seen in Table 1, statin treatment in BDL-cirrhotic rats was associated with hepatic and muscular toxicity. Furthermore, statin treated BDL rats experienced a significant weight loss (from 25–42 g weight loss in treated rats *vs* 2 g in vehicle) compared with vehicles during treatment (Suppl. Table 1). Simvastatin treated BDL rats showed a higher mortality rate (80% at simvastatin 25 mg/kg/day, SIM-25) compared with the other statin treatment groups (from 0 to 6.7%) and the remaining animals presented both muscular and hepatic toxicity (creatine kinase (CK) >1000 IU/L and alanine aminotransferase (ALT) >200 IU/L, respectively). Reduction in simvastatin dose to 10 mg/kg/day decreased mortality rate (18.2%), but high hepatic (66.7%) and muscular (77.8%) toxicity rates were maintained. Although atorvastatin and NCX 6560 treatment did not produce an increment in mortality rates, BDL rats treated with atorvastatin (atorvastatin 10 mg/kg/day, ATO-10; atorvastatin 15 mg/kg/day, ATO-15) and with the highest dose of NCX 6560 (NCX 6560 35.1 mg/kg/day, NCX-35.1) showed some hepatic (14.3%, 6.7% and 20%, respectively) and muscular toxicity (35.7%, 33.3% and 33.3% respectively). However, at equivalent doses of NCX 6560 (NCX 6560 11.7 mg/kg/day, NCX-11.7; NCX 6560 17.5 mg/kg/day, NCX-17.5), hepatic toxicity was eliminated and muscular toxicity reduced. Considering the two equivalent doses (ATO-15, NCX-17.5 and ATO-10, NCX-11.7) the accumulated toxicity (hepatic and/or muscular toxicity) was significantly lower with NCX 6560 treatment, compared with atorvastatin treatment (ATO 37.9% *vs* NCX 11.5%; p = 0.02) (Table 1).

Regarding the CCl_4_ model that mimics a compensated cirrhosis, no mortality events were reported with the different treatments and only animals treated with atorvastatin showed a higher weight loss (10 g vs 3 g in vehicle) and a 7.69% hepatic or muscular toxicity (ALT > 500 IU/L and CK > 6000 IU/L, respectively) (Table 2, Suppl. Table 2). The higher cut-offs for hepatic and muscular toxicity in CCl_4_ rats were due to higher levels of ALT and CK in vehicle treated rats, caused by CCl_4_ and phenobarbital toxicity^33^,^34^.

### Hemodynamic and biochemical changes due to statin treatment in cirrhotic rats

Due to the high mortality and hepatic toxicity rates related to simvastatin treatment in the BDL model, the remaining simvastatin treated rats were not considered in the study and the analysis of their samples and data was not performed.

In this animal model, atorvastatin and NCX 6560 treatment at equivalent doses significantly reduced PP levels (12.2% reduction, p = 0.02, and 12.3% reduction, p = 0.005, for BDL-ATO-15 and BDL-ATO-10, respectively; 14.7% reduction, p = 0.049, and 11.3% reduction, p = 0.026, for BDL-NCX-17.5 and BDL-NCX-11.7, respectively) without changing systemic hemodynamics, compared with BDL vehicles; no significant differences in the PP lowering effect among the different groups were seen. Doubling NCX 6560 dose up to 35.1 mg/kg/day caused an increase in toxicity, without achieving a significant reduction in PP (Table 3).

A similar scenario was observed in CCl_4_-early cirrhotic rats treated with equivalent doses of atorvastatin and NCX-6560 (a non-significant 6.6% reduction in PP for CCl_4_-ATO-15 and a 9.6% reduction for CCl_4_-NCX-17.5) (Table 4).

Suppl. Tables 1 and 2 show the biochemical parameters of blood samples from each group in the two models. Regardless of the treatment group, statin treated cirrhotic rats showed no changes in cholesterol levels. In the BDL model, alkaline phosphatase levels were lower in the treated groups than in BDL vehicle (especially in BDL-ATO-10 and BDL-NCX-35.1), ATO-15 caused a significant increment in total bilirubin levels and treatment with both doses of atorvastatin produced a significant increase in serum creatinine levels with a decrease in urinary volume that was prevented with NCX 6560 treatment. Concerning the CCl_4_ model, rats treated with NCX-17.5 showed significantly increased albumin levels compared to CCl_4_-ATO-15.

### Changes in vasoactive mediators in liver samples from statin treated cirrhotic rats

**Figure 1:**
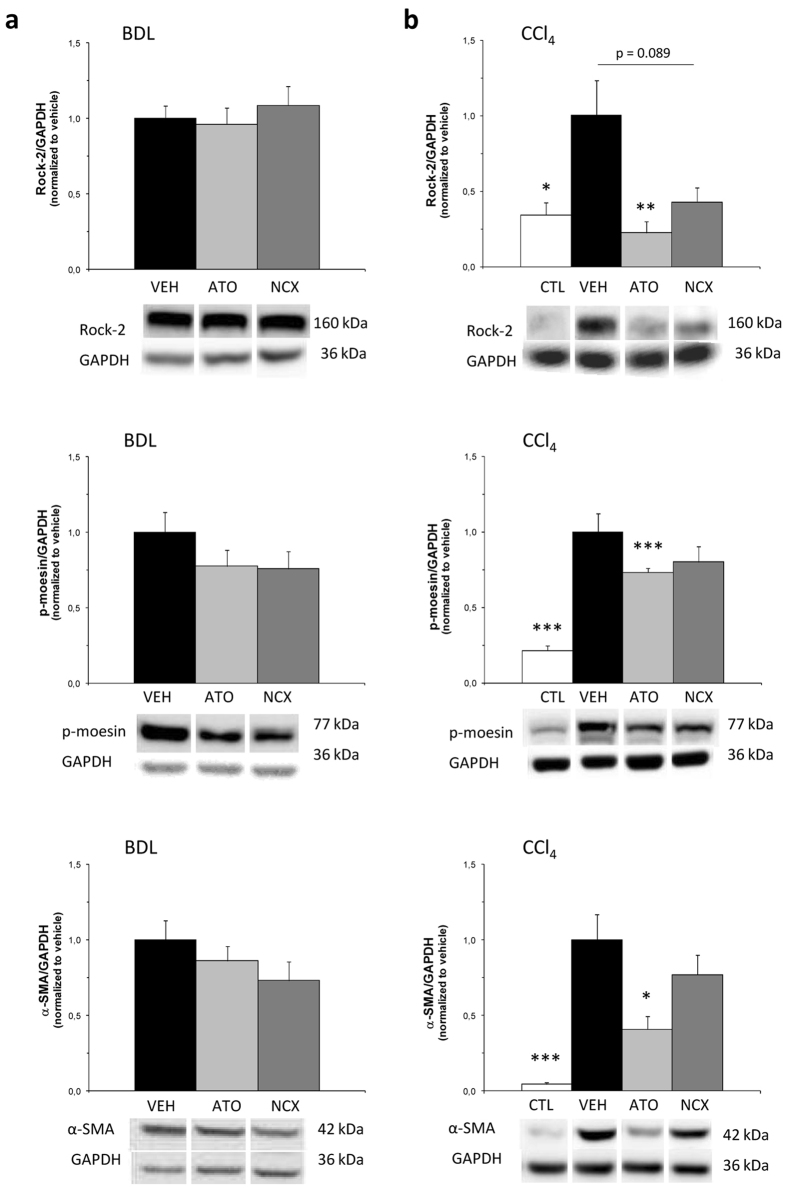
Western blot analysis of intrahepatic markers involved in vasoconstriction and hepatic stellate cell activation. Bar diagrams showing protein quantification of Rho-associated protein kinase 2 (Rock-2), p-moesin and α-smooth muscle actin (α-SMA). Protein levels are expressed as mean ± SEM and normalized to vehicles. Representative Western blots are shown below. GAPDH was used as loading control. (**a**) 4-week bile duct-ligated (BDL) rats after one-week treatment. VEH:vehicle, n = 8; ATO:atorvastatin 15 mg/kg/day, n = 9; NCX:NCX 6560 17.5 mg/kg/day, n = 9. (**b**) Control and 13-week carbon tetrachloride (CCl_4_) rats after a 10-day treatment. CTL:control, n = 7; VEH:vehicle, n = 7; ATO:atorvastatin 15 mg/kg/day, n = 7; NCX:NCX 6560 17.5 mg/kg/day, n = 7. *p ≤ 0.05,**p ≤ 0.01,***p ≤ 0.001 compared with vehicle.

**Figure 2:**
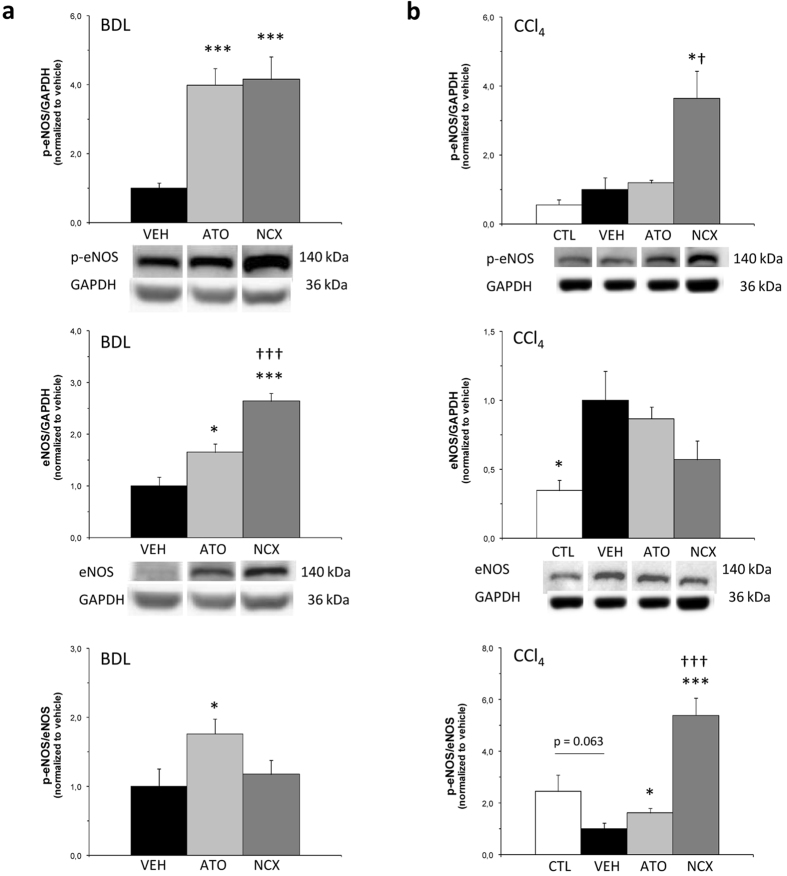
Western blot analysis of intrahepatic eNOS expression. Bar diagrams showing protein quantification of endothelial nitric oxide synthase (eNOS) and p-eNOS. Protein levels are expressed as mean ± SEM and normalized to vehicles. Representative Western blots are shown below. GAPDH was used as loading control. (**a**) 4-week bile duct-ligated (BDL) rats after one-week treatment. VEH:vehicle, n = 8; ATO:atorvastatin 15 mg/kg/day, n = 9; NCX:NCX 6560 17.5 mg/kg/day, n = 9. (**b**) Control and 13-week carbon tetrachloride (CCl_4_) rats after a 10-day treatment. CTL:control, n = 7; VEH:vehicle, n = 7; ATO:atorvastatin 15 mg/kg/day, n = 7; NCX:NCX 6560 17.5 mg/kg/day, n = 7. *p ≤ 0.05, ***p ≤ 0.001 compared with vehicle. ^†^p ≤ 0.05, ^†††^p ≤ 0.001 compared with the equivalent dose of atorvastatin.

**Figure 3:**
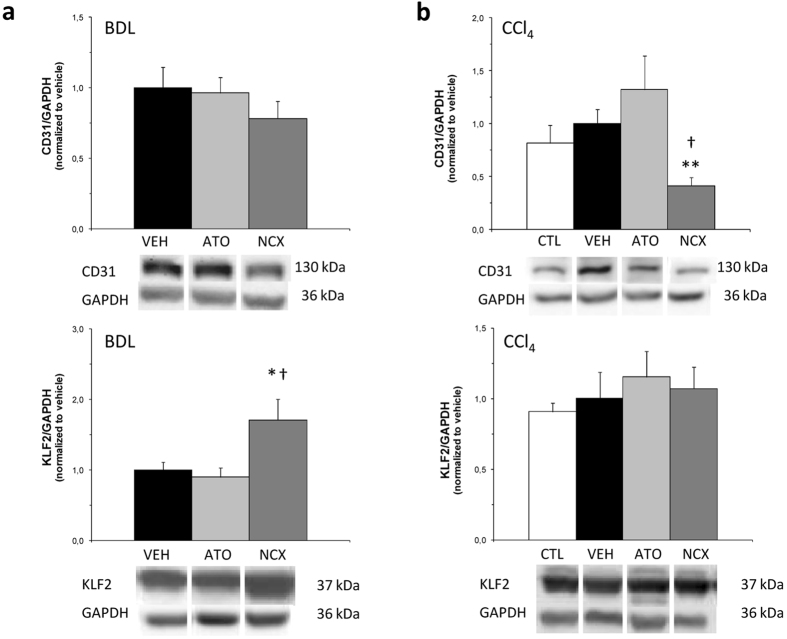
Western blot analysis of intrahepatic markers involved in endothelial dysfunction. Bar diagrams showing protein quantification of CD31 and Krüppel-like factor 2 (KLF2). Protein levels are expressed as mean ± SEM and normalized to vehicles. Representative Western blots are shown below. GAPDH was used as loading control. (**a**) 4-week bile duct-ligated (BDL) rats after one-week treatment. VEH:vehicle, n = 8; ATO:atorvastatin 15 mg/kg/day, n = 9; NCX:NCX 6560 17.5 mg/kg/day, n = 9. (**b**) Control and 13-week carbon tetrachloride (CCl_4_) rats after a 10-day treatment. CTL:control, n = 7; VEH:vehicle, n = 7; ATO:atorvastatin 15 mg/kg/day, n = 7; NCX:NCX 6560 17.5 mg/kg/day, n = 7. *p ≤ 0.05, **p ≤ 0.01 compared with vehicle. ^†^p ≤ 0.05, compared with the equivalent dose of atorvastatin.

ATO-15 and the equivalent dose of NCX 6560 caused no changes in Rho-associated protein kinase 2 (Rock-2) protein expression in BDL cirrhotic rats compared with BDL vehicle, but Rho-kinase activity, assessed as the phosphorylation of the endogenous Rho-kinase substrate, moesin, at Thr-558, was slightly lower, although not significantly. In CCl_4_-cirrhotic rats both the expression of Rock-2 and its activity decreased in CCl_4_-ATO-15 and CCl_4_-NCX-17.5 treatment groups (Fig. 1). Both treatments with atorvastatin and NCX 6560 in the BDL model not only caused a significant increment in p-eNOS, but also in total eNOS. This increase in eNOS protein levels was much higher in the BDL-NCX 6560 group, being significantly different from BDL-atorvastatin total eNOS levels. By contrast, in the CCl_4_ model an increase in the p-eNOS/eNOS ratio was observed with both treatments, being much higher in the CCl_4_-NCX 6560 group (Fig. 2). A mild non-significant decrease in the expression of the platelet/endothelial cell adhesion molecule-1 (CD31/PECAM-1) with NCX-17.5 treatment was also observed in the BDL model, while in the CCl_4_ model this reduction was significant. *De novo* expression of CD31 has been used to reflect endothelial dysfunction^35^,^36^ and the decrease observed here suggests an improvement in the endothelial phenotype. Regarding the expression of KLF2, we also observed a significant increment in its expression in BDL rats treated with NCX-17.5 compared with BDL-VEH and BDL-ATO-15 treated groups (Fig. 3a).

### Liver fibrosis, inflammation and liver cyclic guanosine monophosphate (cGMP) levels in statin treated cirrhotic rats

**Figure 4:**
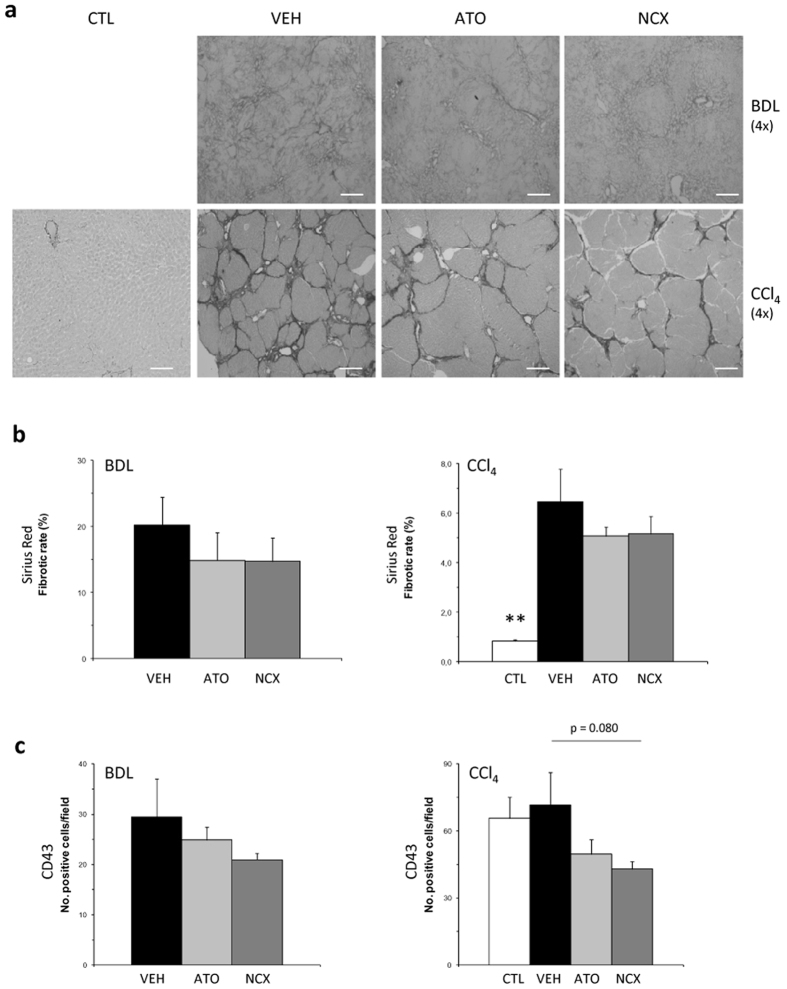
Histologic assessment of liver fibrosis and inflammation. (**a**) Representative images of liver sections stained by Sirius Red from 4-week bile duct-ligated (BDL) rats or 13-week carbon tetrachloride (CCl_4_) rats treated with statins or vehicle and from control rats (original magnification: 4x [white scale bar = 200 μm]). (**b**) Bar diagram showing Sirius Red quantification expressed as fibrotic rate (%). (**c**) Bar diagrams showing CD43 immunohistochemistry quantification in livers from 4-week BDL rats and 13-week CCl_4_ rats treated with statins or vehicle and from control rats. VEH:vehicle (BDL: n = 8, CCl_4_: n = 7); ATO:atorvastatin 15 mg/kg/day (BDL: n = 9, CCl_4_: n = 7); NCX:NCX 6560 17.5 mg/kg/day (BDL: n = 9, CCl_4_: n = 7); CTL:controls (n = 7).**p ≤ 0.01 compared with vehicle.

A reduction in alpha-smooth muscle actin (α-SMA) protein expression with both atorvastatin and NCX 6560 treatments was observed in both models, showing a more prominent and significant decrease in the CCl_4_ model (Fig. 1). Sirius Red stained liver sections from 4-week BDL rats and 13-week CCl_4_ and control rats treated with VEH, ATO-15 and NCX-17.5 are shown in Fig. 4a. Although not significant, both atorvastatin and NCX 6560 treatments caused a reduction in the red-stained area per total area, suggesting a trend towards a decrease in fibrillar collagen content in both models (Fig. 4b). In addition, both atorvastatin and NCX 6560 treatments also increased liver tissue cGMP content, a marker of NO bioavailability in the BDL cirrhotic liver. Hepatic cGMP levels in BDL animals treated with ATO-15 (1.91 ± 0.33 pmol/(mL.100 mg)) and with NCX-17.5 (1.42 ± 0.25 pmol/(mL.100 mg)) were significantly higher than in BDL-VEH (0.74 ± 0.05 pmol/(mL.100 mg); p = 0.002 and p = 0.016, respectively) and no significant differences between BDL-ATO-15 and BDL-NCX-17.5 were seen (p = 0.259). Moreover, there was a non-significant decrease in the number of leukosialin (CD43) immunostained cells in liver sections from animals treated with ATO-15 and NCX-17.5 in both models, suggesting a lower hepatic inflammatory state (Fig. 4c).

## Discussion

Statins are progressively becoming a focus of attention as a potential new therapy for chronic liver disease^37^. Indirect evidences from epidemiological studies indicate that statin use is associated with a decreased risk of fibrosis progression, cirrhosis, hepatic decompensation, hepatocellular carcinoma and death in patients with chronic liver disease, especially with hepatitis C virus infection^38^,^39^,^40^,^41^,^42^. More direct evidences from clinical trials point to a decreased mortality in decompensated cirrhotic patients receiving statins^27^. However, the mechanisms involved in these effects are not well known, differences among statins and doses have not been assessed and more importantly, concerns about the safety of statins in decompensated cirrhotic patients exist.

Our results in cirrhotic rats confirm previous findings: a trend (although not significant) towards a reduction in PP after statin treatment in CCl_4_-intoxicated rats^10^,^28^ and a significant decrease in PP in BDL rats^11^,^43^, without changing systemic hemodynamics. Unfortunately, we were not able to evaluate systemic and portal hemodynamics with simvastatin treatment, not even after reducing the statin dose to 10 mg/kg/day, due to its high mortality and hepatic toxicity in the BDL model. Almost two decades ago, the first experiments with simvastatin were performed by Oberti and colleagues^44^. BDL rats treated with simvastatin 2.5 mg/kg/day over a 4-week period from the beginning showed neither hepatic toxicity nor therapeutic benefit on hemodynamics and liver fibrosis. Subsequently, two more groups described effects in PP with 3-day simvastatin 25 mg/kg/day treatment in CCl_4_-cirrhotic rats and a decrease in portal-systemic collateral vascular resistance and PP in partially portal vein-ligated rats receiving simvastatin 20 mg/kg/day for 9 days, but no data on liver function tests were given^10^,^45^. Similar doses (25 mg/kg/day) have been also administered in non cirrhotic rats showing data of a hepatoprotective activity of simvastatin in endotoxemia^46^. Differences among the results in these studies might be attributed to different treatments and animal models. However, it is clear that at high doses (10–25 mg/kg/day) and for 7 or more days, the rat BDL model is not suitable to study simvastatin effects on liver cirrhosis, probably due to the accumulation of active metabolites in the liver unable to be cleared through biliary excretion^47^.

Although not in the same magnitude, atorvastatin treatment is also associated with some muscular and hepatic toxicity in the BDL model and with lower toxicity rates in the CCl_4_ model. Specifically in our study, we ascribe the differences in toxicity to the contrast between the two models: while the effect of statins in an early cirrhotic state is tested with the CCl_4_ model, the BDL model causes a more aggressive, cholestatic injury that together with the fact that the model itself impairs drug clearance mimics a severely deteriorated liver function. Thus, the higher toxicity rates observed with statin treatment in the BDL model reinforce the idea that despite their beneficial effects in liver cirrhosis, caution when prescribing statins is required, in patients with deteriorated liver function, who might develop rhabdomyolysis at lower doses than the general population^27^.

Both simvastatin and atorvastatin induce a similar adaptive response in cells, their actions are qualitatively and mechanistically identical and the main difference between them is their pharmacokinetics^48^. Considering that the equipotent dose of simvastatin and atorvastatin in humans is about 2:1, since plasma is cleared of atorvastatin more slowly than it is of simvastatin^49^,^50^, and that both drugs are mainly eliminated through biliary excretion^47^, apparently there is no clear explanation for the high differences in toxicity that we observed in BDL cirrhotic rats. However, a study of the mechanisms involved in statins cytotoxicity, mainly through oxidative stress, in freshly isolated rat hepatocytes showed that simvastatin was the most cytotoxic statin^51^. Besides, statin drug-induced liver injury in humans is rare, but can be associated with severe outcomes^16^,^17^,^18^, and safety data from statin clinical trials should be interpreted with caution given that they normally exclude patients with advanced liver failure, although current evidences indicate that tolerability is good even in patients with liver cirrhosis^27^.

For all these reasons, NCX 6560, a NO-donating atorvastatin, could be a safer alternative to treating cirrhotic patients with portal hypertension. Our results in BDL and CCl_4_ rats prove that this drug achieves an equivalent atorvastatin PP lowering effect without affecting systemic hemodynamics. NCX 6560 not only improves statin-induced myopathy^31^, but also prevents hepatic toxicity caused by equivalent doses of atorvastatin, probably due to its greater anti-inflammatory and antioxidant properties^29^,^32^. However, higher doses of NCX 6560 (35.1 mg/kg/day) were not associated with any significant additional PP lowering effect, while toxicity increased moderately. Similarly, in atorvastatin treated animals there was no direct correlation between higher doses and more hemodynamic effects. Indeed, dose-dependency of the pleiotropic effects of atorvastatin treatment remains unclear and differs among individual biological effects^52^. In addition, we speculate that the cytotoxicity toward hepatocytes caused by statins^51^, such as cell death, reactive oxygen species formation or lipid peroxidation, among others, could mask, in a dose dependent manner, its beneficial effects on IHVR and ultimately on portal pressure.

By measuring SMABF, a surrogate of portal blood inflow, we show that NCX 6560 does not enhance splanchnic vasodilation of cirrhosis, which together with the lack of changes in mean arterial pressure (MAP), suggests that NCX 6560 effects are liver-selective. Moreover, impairment of systemic hemodynamics and the risk of renal failure, the main drawback of NO donors^7^, seems to be prevented, since diuresis from NCX 6560 treated cirrhotic rats was not decreased and serum creatinine levels were maintained compared with vehicles, whereas atorvastatin treatment reduced urinary volume in both models and even significantly increased serum creatinine levels in the BDL model, compared with vehicles. Given that serum creatinine levels in atorvastatin treated animals with muscular toxicity were significantly higher than in animals without it (data not shown), the higher incidence of muscular toxicity due to atorvastatin treatment could be related to kidney failure. In addition, NCX 6560 also improved albumin levels in the CCl_4_ model, compared with the atorvastatin group.

No changes in serum cholesterol levels were seen among the different groups in the two models. Therefore, the advantages of atorvastatin and NCX 6560 in liver hemodynamics must be caused by the so-called pleiotropic effects of statins^19^,^20^,^21^. According to hemodynamic results, both atorvastatin and NCX 6560 seemed to have similar beneficial intrahepatic effects in the two models: they slightly reduced the liver fibrotic area, lowered the phosphorylated moesin (p-moesin) expression and significantly increased p-eNOS, compared with vehicles, confirming previous results with atorvastatin^11^. This was associated with an increase in hepatic cGMP, the second messenger of NO, indicating an improvement in NO availability, probably due to a decrease in oxidative stress related to statin therapy^19^. Although we expected higher cGMP levels in rats treated with NCX 6560, no significant differences between BDL-ATO-15 and BDL-NCX-17.5 were observed, which is in line with the fact that NCX 6560 neither exerted a greater PP lowering effect nor a higher reduction in Rho-kinase activity than atorvastatin. This suggests that the improvement in toxicity seen in our model with the NO-donating drug might be independent from the cGMP signaling pathway.

Additionally, NCX 6560 tended to have a slightly better anti-inflammatory effect in the two models, which could partially explain its lower hepatic toxicity. Moreover, NCX 6560 seemed to have a more pronounced beneficial intrahepatic effect because it significantly increased KLF2 and eNOS protein expression compared with the atorvastatin group in the BDL model, and the p-eNOS/eNOS ratio was much higher than in the atorvastatin group in the CCl_4_ model. These effects could also be contributing to its lower hepatic toxicity, improving the endothelial phenotype, as seen by the decrease of CD31, especially in the CCl_4_ model, and therefore, to hepatocytes viability.

Another important factor contributing to increase IHVR in cirrhosis is hepatic stellate cell activation. The transdifferentiation process of hepatic stellate cells into myofibroblasts with contractile, pro-inflammatory and fibrogenic properties, involves expression of α-SMA and cytoskeleton reorganization with loss of lipid droplets^53^. We also observed a decrease in α-SMA protein expression, more prominent in the CCl_4_ model, in livers from statin treated rats compared with vehicles. Apart from conferring hepatic vasoprotection, KLF2 also promotes stellate cells deactivation through a KLF2-NO-guanylate cyclase paracrine mechanism^26^,^54^. The high KLF2 expression seen in livers from NCX 6560 treated BDL rats could contribute to the significant increment in eNOS in this treatment group, without a significant decrease in the vasoconstrictive pathway (RhoA, Rho-kinase) responsible for the inhibition of eNOS^25^,^55^. Neither differences in KLF2 expression in livers from atorvastatin treated BDL rats compared with vehicles, nor significant increments with statin treatment in the CCl_4_ model were observed. Marrone and colleagues^26^ showed that atorvastatin was the less effective, among the statins tested, in inducing KLF2 mRNA expression in sinusoidal endothelial cells. Moreover, Gracia-Sancho and colleagues^25^ showed that in a more aggressive model of CCl_4_ inhalation three times a week in 50–75 g animals, KLF2 expression was starting to be induced at 6 weeks and continue increasing during the progression of cirrhosis. The 6-week model of Gracia-Sancho is probably equivalent to our 13-week model, since at least PP values (around 9 mmHg) are the same. The disparity of our results between BDL and CCl_4_ models could be attributed to differences in the induction of cirrhosis and mainly to the stage of liver disease; while in the BDL model KLF2 expression is highly induced, in our CCl_4_ model it is not initiated yet.

In summary, conventional statins ameliorate portal hypertension, but their adverse events are magnified in a model that mimics a deteriorated liver function. By contrast, NCX 6560 has similar effects in the two cirrhotic models with a safer toxicity profile compared with conventional statins. Additionally, due to its liver NO release, it induces a higher intrahepatic vasoprotective profile that might have a more long-term beneficial effect in the intrahepatic vascular alterations of portal hypertension than conventional statins. Also, in general terms, the intrahepatic improvements of NCX 6560 treatment were greater in the CCl_4_ model, reinforcing a major benefit of statins when given earlier in the development of cirrhosis and for longer periods. These results suggest that NCX 6560 could be a safer option for long-term statin treatment of portal hypertension in cirrhotic patients.

## Material and Methods

### Experimental design

Two different approaches were designed in which statins were evaluated:

1. Four-week BDL rats: a cirrhotic model that simulates a decompensated chronic liver disease in order to compare the efficacy and toxicity among statins (7 days) and establish the appropriate dose.
2. Thirteen-week CCl_4_-treated rats: a model of early cirrhosis in order to evaluate their beneficial intrahepatic effects after a longer treatment period (10 days).

### Experimental models of cirrhosis

In the decompensated chronic liver disease model, cirrhosis was induced by BDL. Male Sprague-Dawley OFA rats (Charles River Laboratories, L’Arbresle, France) weighing 200–220 g were anaesthetized with inhaled isoflurane and the common bile duct was occluded by double ligature with a 4–0 silk thread. The bile duct was then resected between the two ligatures. Animals received weekly intramuscular vitamin k1 to decrease mortality from bleeding^56^.

To obtain an early cirrhosis model, male Wistar rats (Charles River Laboratories, L’Arbresle, France) weighing 100–120 g followed a CCl_4_ inhalation protocol^43^ for 13 weeks. Phenobarbital (0.3 g/L) was added to drinking water one week before the inhalation protocol.

All rats were kept under constant temperature and humidity in a 12 h controlled dark/light cycle and fed *ad libitum* with a grain-based chow (Teklad 2014, Harlan Laboratories, Indianapolis, IN, USA) containing a fixed formula of ingredients with 0.1% Na^+^.

### Drug administration

Four-week BDL rats received daily (q.d.) oral doses of statins or vehicle for 7 days (treatment started on the beginning of the fourth week). Two doses of each statin were evaluated: simvastatin (Ratiopharm, Madrid, Spain) 25 mg/kg/day (SIM-25, n = 10) and 10 mg/kg/day (SIM-10, n = 11), atorvastatin (Almirall, Barcelona, Spain) 15 mg/kg/day (ATO-15, n = 14) and 10 mg/kg/day (ATO-10, n = 15) and NCX 6560 ((βR, δR)-2(4-fluorophenyl)-β, δ-dihydroxy-5-(1-methylethyl)-3-phenyl-4-[(phenylamino) carbonyl]-1H-pyrrole-1heptanoic acid 4-(nitrooxy)butyl ester) (NicOx S.A., Sophia Antipolis, France) 17.5 mg/kg/day (NCX-17.5, n = 11) and 11.7 mg/kg/day (NCX-11.7, n = 15). An additional dose of NCX 6560 at 35.1 mg/kg/day (NCX-35.1, n = 15) was also tested and polyethylene glycol (PEG) 70% was used as a vehicle (VEH, n = 12). Starting dosage was chosen according to previous literature^10^,^11^,^23^,^43^ and the equivalent doses of NCX 6560 and atorvastatin used were calculated on the basis of their different molecular weights (MW NCX 6560/MW atorvastatin: ratio 1.17) and contained the same amount of atorvastatin: ATO-15 equals to NCX-17.5 and ATO-10 to NCX-11.7.

In the CCl_4_-cirrhotic model, the treatments (ATO-15, n = 13, NCX-17.5, n = 13 and VEH, n = 8) were given orally (q.d.) for 10 days and began at the beginning of the thirteenth week of CCl_4_ inhalation. CCl_4_ inhalation was kept during treatment to mimic a continuous and chronic liver injury, but last inhalation session was at least 3 days before ending treatment. Control rats (CTL, n = 8) received phenobarbital in drinking water, but neither followed the CCl_4_ inhalation protocol nor received any treatment.

All treatments in the different studies were administered by gastric gavage. During treatment rats were weighed daily and experiments were performed ninety minutes after the last dose of statin or vehicle.

### Hemodynamic measurements

Rats were anaesthetized for continuous measurement of MAP (mmHg), PP (mmHg), superior mesenteric artery (SMA) blood flow (SMABF, mL/[min.100 g]) and portal blood flow (PBF, mL/[min.100 g]). SMA resistance (SMAR, mmHg/mL.min.100 g) was calculated as ([MAP-PP]/SMABF) and IHVR as (PP/PBF) (see details in Supplementary material).

### Sample collection

For the determination of the diuresis volume, urine was collected from the bladder at the end of the 1-hour period of hemodynamic registration. Venous blood samples were obtained from the cava vein after the hemodynamic measurements. Liver from cirrhotic rats was perfused with saline for exsanguination and cut into fragments. Liver samples were either snap-frozen in liquid nitrogen and stored at −80 °C or fixed in 4% formaldehyde solution for 24 h and changed to 50% ethanol solution before paraffin embedding.

### Biochemical parameters

Serum levels of sodium, potassium, osmolality, creatinine, total bilirubin, ALT and aspartate aminotransferase (AST), alkaline phosphatase (ALP), CK, albumin and cholesterol were determined using an automatic analyzer (Olympus AV5400, Olympus Europe GmbH, Hamburg, Germany).

Hepatic and muscular toxicity due to statin treatment was defined based on ALT and CK levels in vehicle rats from both models. Thus, we defined hepatic toxicity as an increment in ALT levels superior to 200 IU/L (BDL model) and to 500 IU/L (CCl_4_ model), whereas muscular toxicity was considered when CK levels were above 1000 IU/L (BDL model) and 6000 IU/L (CCl_4_ model). Animals with hepatic toxicity due to statin treatment were discarded from the study and were not used for sample and data analysis.

### Western blot analysis

Protein extraction from liver samples and immunoblotting was performed as previously described^10^ (see details in Supplementary material). The following primary antibodies were used: α-SMA (dil. 1/200) (Abcam, Cambridge, UK), glyceraldehyde-3-phosphate dehydrogenase (GAPDH, dil. 1/5000) (Ambion, Austin, TX, USA), eNOS (dil. 1/500) (BD Transduction Laboratories, Lexington, KY, USA), phosphorylated eNOS (p-eNOS, Ser1177, dil. 1/250), KLF2 (N-13, dil. 1/200), CD31/PECAM-1 (M-20, dil. 1/200), p-moesin (Thr 558, dil. 1/200) and Rock-2 (H-85, dil. 1/200) (Santa Cruz Biotechnology, Dallas, TX, USA).

### Statistical analysis

All values are expressed as mean ± standard error of the mean (SEM) and compared with vehicles using Student’s *t* test (SigmaStat 3.0) or ANOVA test, when indicated. For the toxicity analysis, Fisher Exact test for contingency tables were used. Statistical significance was established at p ≤ 0.05.

### Ethic statement

All animals received humane care in accordance with institutional guidelines from the European Commission on the protection of animals used for experimental and other scientific purposes (Directive 2010/63/EU). All experiments were approved by the Animal Care Committee of the Vall d’Hebron Institut de Recerca (VHIR, Barcelona, Spain) (DAAM Permission No.: 7834) and conducted in the animal facilities of VHIR.

See Supplementary material for details on Sirius Red staining, immunohistochemistry and cGMP determination.

## Additional Information

**How to cite this article**: Rodríguez, S. *et al*. A Nitric Oxide-Donating Statin Decreases Portal Pressure with a Better Toxicity Profile than Conventional Statins in Cirrhotic Rats. *Sci. Rep.* **7**, 40461; doi: 10.1038/srep40461 (2017).

**Publisher's note:** Springer Nature remains neutral with regard to jurisdictional claims in published maps and institutional affiliations.

## Supplementary Material

Supplementary Material

## Figures and Tables

**Table 1:** Adverse events after one week treatment in 4-week bile duct-ligated rats.

	Dose (mg/kg/day)	n	Mortality rate (%)	Hepatic toxicity rate (%)	Muscular toxicity rate (%)
**Vehicle**		12	16.7	0	0
**Simvastatin**	25	10	80	100	100
	10	11	18.2	66.7	77.8
**Atorvastatin**	15	14	0	14.3	35.7
	10	15	6.7	6.7	33.3
**NCX 6560**	35.1	15	6.7	20	33.3
	17.5	11	0	0	9.1
	11.7	15	0	0	13.3

n, number of rats that initiated the treatment protocol; Mortality rate, % of animals that died during the experimental protocol (through treatment or anesthesia); Hepatic toxicity rate, % of animals with serum ALT levels >200 IU/L; Muscular toxicity rate, % of animals with serum CK levels >1000 IU/L. Equivalent doses: NCX 6560 17.5 mg/kg/day equals to atorvastatin 15 mg/kg/day and NCX 6560 11.7 mg/kg/day equals to atorvastatin 10 mg/kg/day.

**Table 2:** Adverse events in control rats and in 13-week CCl_4_-induced cirrhotic rats after a 10-day treatment.

	Dose (mg/kg/day)	n	Mortality rate (%)	Hepatic toxicity rate (%)	Muscular toxicity rate (%)
**CCl**_**4**_**-vehicle**		8	0	0	0
**CCl**_**4**_**-atorvastatin**	15	13	0	7.69	7.69
**CCl**_**4**_**-NCX 6560**	17.5	13	0	0	0
**Control**		8	0	0	0

CCl_4_, cirrhotic rats induced by carbon tetrachloride; n, number of rats that initiated the treatment protocol; Mortality rate, % of animals that died during the experimental protocol (through treatment or anesthesia); Hepatic toxicity rate, % of animals with serum ALT levels >500 IU/L; Muscular toxicity rate, % of animals with serum CK levels >6000 IU/L. Equivalent doses: NCX 6560 17.5 mg/kg/day equals to atorvastatin 15 mg/kg/day.

**Table 3:** Hemodynamic measurements in 4-week bile duct-ligated rats after one-week treatment (values taken 2 h 30 min after the last dose of treatment).

	Dose (mg/kg/day)	n	MAP (mmHg)	PP (mmHg)	SMABF (mL/[min·100 g])	SMAR (mmHg/mL·min·100 g)	Heart rate (bpm)
**Vehicle**		8	96.39 ± 6.84	18.53 ± 0.56	4.42 ± 0.31	17.99 ± 1.73	318.10 ± 12.88
**Atorvastatin**	15	9	82.46 ± 4.44	16.27 ± 0.67*	4.98 ± 0.41	13.95 ± 1.34	314.82 ± 9.96
	10	11	84.11 ± 4.22	15.77 ± 0.59**	3.63 ± 0.26	19.60 ± 1.59	314.65 ± 12.75
**NCX 6560**	35.1	9	105.83 ± 5.11	17.75 ± 0.64	4.30 ± 0.41	22.10 ± 2.43	327.77 ± 15.92
	17.5	9	85.35 ± 5.95	16.25 ± 0.86*	3.88 ± 0.37	19.24 ± 2.22	310.84 ± 13.40
	11.7	9	95.67 ± 6.42	16.43 ± 0.63*	3.91 ± 0.41	22.17 ± 3.18	330.51 ± 9.05

Values are expressed as mean ± SEM. n, number of rats; MAP, mean arterial pressure; PP, portal pressure; SMABF, superior mesenteric artery blood flow; SMAR, superior mesenteric artery resistance. Equivalent doses: NCX 6560 17.5 mg/kg/day equals to atorvastatin 15 mg/kg/day and NCX 6560 11.7 mg/kg/day equals to atorvastatin 10 mg/kg/day. *p ≤ 0.05, **p ≤ 0.01 compared with vehicle.

**Table 4:** Hemodynamic measurements in control rats and in 13-week CCl_4_-induced cirrhotic rats after a 10-day treatment.

	CCl_4_-vehicle	CCl_4_-atorvastatin(15 mg/kg/day)	CCl_4_-NCX 6560(17.5 mg/kg/day)	Control
**n**	8	11	13	7
**MAP (mmHg)**	81,56 ± 6,62	85,52 ± 2,69	81,73 ± 3,65	86,46 ± 7,74
**PP (mmHg)**	9,39 ± 0,62	8,77 ± 0,42	8,49 ± 0,31	7,59 ± 0,44*
**SMABF (mL/[min·100 g])**	2,85 ± 0,56	2,23 ± 0,34	2,32 ± 0,31	1,63 ± 0,23
**SMAR (mmHg/mL·min·100 g)**	28,51 ± 4,42	39,86 ± 4,26	36,28 ± 3,89	51,90 ± 6,61*
**Heart rate (bpm)**	318,28 ± 18,21	360,31 ± 17,86	307,27 ± 12,50	318,16 ± 17,31
**PBF (mL/[min·100 g])**	1,95 ± 0,26	2,08 ± 0,35	2,10 ± 0,21	1,66 ± 0,27
**IHVR (mmHg/mL·min·100 g)**	5,61 ± 0,96	5,02 ± 0,66	4,32 ± 0,42	5,02 ± 0,74

Values are expressed as mean ± SEM. CCl_4_, cirrhotic rats induced by carbon tetrachloride; n, number of rats; MAP, mean arterial pressure; PP, portal pressure; SMABF, superior mesenteric artery blood flow; SMAR, superior mesenteric artery resistance; PBF, portal blood flow; IHVR, intrahepatic vascular resistance. *p ≤ 0.05 compared with CCl_4_-vehicle.
