# Supplementary material for: A Nitric Oxide-Donating Statin Decreases Portal Pressure with a Better Toxicity Profile than Conventional Statins in Cirrhotic Rats
Source: Sci Rep. 2017 Jan 13;7:40461. doi: 10.1038/srep40461 (PMC5233977; doi:10.1038/srep40461)
Supplement: Supplementary Material [file srep40461-s1.doc]

**Supplementary material to:**

**A NITRIC OXIDE-DONATING STATIN DECREASES PORTAL PRESSURE WITH A BETTER TOXICITY PROFILE THAN CONVENTIONAL STATINS IN CIRRHOTIC RATS**

Sarai Rodríguez^1^, Imma Raurell^1,2^, Manuel Torres^1^, Teresa García-Lezana^1,2^, Joan Genescà^1,2^, María Martell^1,2^.

^1^ Liver Diseases Laboratory, Liver Unit, Department of Internal Medicine, Hospital Universitari Vall d’Hebron, Institut de Recerca (VHIR), Universitat Autònoma de Barcelona, Barcelona, Spain.

^2^ Centro de Investigación Biomédica en Red de Enfermedades Hepáticas y Digestivas (CIBERehd), Instituto de Salud Carlos III, Madrid, Spain.

**TABLE OF CONTENTS**

Page

**Methods** 2

*Hemodynamic measurements* 2

*Western blot analysis* 3

*Sirius Red staining* 3

*Immunohistochemistry* 4

*Liver cGMP concentration* 4

**Supplementary tables** 6

*Supplementary table 1* 6

*Supplementary table 2* 7

**Methods**

*Hemodynamic measurements*

Ninety minutes after statin or vehicle administration, 16-hour fasted rats were anaesthetized with ketamine hydrochloride (100 mg/kg) plus midazolam (5 mg/kg) intraperitoneally. Dose adjustments in anaesthesia were made depending on animal’s condition and the treatment received. One polyethylene PE-catheter (PE50) was introduced into the femoral artery to MAP (mmHg) and after a midline abdominal incision, another catheter was introduced into the ileocolic vein for PP measurement (mmHg) using highly sensitive pressure transducers (Harvard Apparatus, Holliston, MA, USA). The SMA was isolated from connective tissue and a perivascular ultrasonic transit-time flowprobe (1mm diameter, Transonic Systems Inc., Ithaca, NY, USA) was placed around the artery to continuously measure the SMABF (mL/[min.100 g]). SMAR (mmHg/mL.min.100 g) was calculated as ([MAP-PP]/SMABF). The same flowprobe was placed on the dissected portal vein to measure portal blood flow (PBF, mL/[min.100 g]) and IHVR was calculated as (PP/PBF).

Ascites volume was determined and body weight calculated as (rat weight - ascites volume) considering 1 mL = 1 g. Animals were maintained at 37 ºC throughout the study by a rectal temperature probe.

Hemodynamic parameters were allowed to equilibrate and measures were obtained 2 h 30 min after the last dose of statin or vehicle (cirrhotic animals) or 1 h after manipulation (control rats), each value representing the average of 30 seconds. Animals were euthanized by exsanguination under anaesthesia.

*Western blot analysis*

For whole protein extraction samples of snap-frozen livers were crushed to powder while frozen and subsequently homogenized in Triton-lysis buffer (25.4 mM Tris/HCl pH 7.6, 137 mM NaCl, 2.7 mM KCl, 20 mM NaF, 10 mM Na_4_P_2_O_7_, 10 nM okadaic acid, 2 mM Na_3_VO_4_, 2 μg/mL antipain, 2 μg/mL aprotinin, 2 μg/mL chymostatin, 2 μg/mL leupeptin, 2 μg/mL pepstatin A, 2 μg/mL trypsin inhibitor, 40 μg/mL phenylmethylsulfonylfluoride, and 10 % v/v Triton X-100). Thereafter they were sonicated (3 x 10 s), left on ice for 10 min, and centrifuged at 4 ºC and 18,000 *g* for 10 min. Supernatant protein concentration was assessed by BCA ™ Protein Assay Kit (Thermo Fisher Scientific, Rockford, IL, USA). Equal amounts of protein (30-70 μg of protein/lane) were run on a 4-12 % or 10 % sodium dodecyl sulphate-polyacrylamide gel electrophoresis (SDS-PAGE) depending on the protein molecular weight. Proteins were blotted onto a polyvinylidene difluoride (PVDF) membrane (Thermo Fisher Scientific, Waltham, MA USA) and membranes were blocked and incubated with primary antibodies and thereafter with corresponding secondary peroxidase-coupled antibody. GAPDH served as endogenous control. Blots were developed with enhanced chemioluminiscence (Amersham ECL Prime, GE Healthcare, Uppsala, Sweden) and protein expression was determined by densitometric analysis using Quantity One software (Bio-Rad Laboratories, Hercules, CA, USA).

*Sirius Red staining*

Liver fibrosis was assessed in 4μm sections from paraffin embedded liver samples. To detect collagen fibers, liver sections were deparaffinised, rehydrated, and stained with 0.1 % Picro-Sirius Red (Direct Red 80 in saturated aqueous picric acid [Sigma-Aldrich, Saint Louis, MO, USA]). The fibrotic area was then assessed using image analysis techniques. Briefly, ten fields (10x magnification) from each Sirius Red stained section were randomly obtained using an optical microscope Olympus BX61 (Olympus, Hamburg, Germany) equipped with a digital camera (large bile ducts and vessels excluded) and the red-stained area per total area was measured using ImageJ 1.38 free software (National Institute of Health, Bethesda, MD, USA) and expressed as fibrotic rate (%).

*Immunohistochemistry*

Liver inflammation was assessed by anti-CD43 (a pan-leukocyte marker) immuhistochemistry in 4μm sections from paraffin embedded liver samples. Briefly, liver sections were deparaffinised, rehydrated, blocked with 1/20 dilution of goat serum and incubated overnight with primary antibody against CD43 (W3/13, dil. 1/500, AbD Serotec Ltd, Oxford, UK). Bound antibody was incubated with ENVISION-HRP anti-mouse secondary antibody (Dako, Glostrup, Denmark), visualized with the VIP substrate kit (Vector. Burlingame, CA, USA) that produces a purple precipitate and counterstained with hematoxylin. Ten fields per section were randomly captured at 10x magnification and images were quantified with the manual cell counter of Image J 1.38 free software (National Institute of Health, Bethesda, MD, USA) obtaining the number of CD43 positive cells per field.

*Liver cyclic guanosine monophosphate (cGMP) concentration*

Measurements of cGMP, a marker of NO bioavailability, were performed in liver homogenates from VEH, ATO-15 and NCX-17.5 treated animals without hepatic toxicity (n=7, n=5 and n=6, respectively). Samples of frozen tissue were crushed to powder and aliquots from each sample containing 100-200 mg of tissue dropped into 5 volumes of 5 % trichloroacetic acid and homogenized on ice. The precipitate was removed by centrifugation at 1500 *g* for 10 min at 4 ºC and the supernatant transferred to a clean test tube, washed five times with five volumes of water-saturated diethyl ether and the aqueous phase extract lyophilized. The dried extract was dissolved in ultrapure water and cGMP levels were determined by enzyme immunoassay (Cayman Chemical Co., Ann Arbor, MI, USA). Results were expressed as pmol/(mL.100 mg).

**Supplementary tables**

**Supplementary table 1**. Characteristics and biochemical parameters of 4-week bile duct-ligated rats after one-week treatment.

|  | **Vehicle** | **Atorvastatin**  **(15 mg/kg/day)** | **Atorvastatin**  **(10 mg/kg/day)** | **NCX 6560**  **(35.1 mg/kg/day)** | **NCX 6560**  **(17.5 mg/kg/day)** | **NCX 6560**  **(11.7 mg/kg/day)** |
| --- | --- | --- | --- | --- | --- | --- |
| **n** | 8 | 9 | 11 | 9 | 9 | 9 |
| **Body weight (g)** | 331.10 ± 9.86 | 313.78 ± 20.55 | 278.21 ± 11.07 ** | 297.47 ± 12.10 | 300.99 ± 10.54 | 305.26 ± 9.25 |
| **Weight loss during treatment (g)** | 2.13 ± 3.54 | 41.59 ± 6.03 *** | 34.93 ± 6.39 *** | 42.30 ± 5.36 *** | 25.70 ± 5.31 ** | 29.01 ± 4.45 *** |
| **Urinary volume (mL/h)** | 0.71 ± 0.17 | 0.34 ± 0.08 | 0.21 ± 0.07 ** | 0.99 ± 0.15 | 0.76 ± 0.15 † | 0.69 ± 0.15 †† |
| **Serum Na^+^ (mmol/L)** | 142.10 ± 1.44 | 140.81 ± 0.43 | 140.56 ± 0.70 | 141.34 ± 0.82 | 141.20 ± 0.74 | 141.30 ± 0.98 |
| **Serum K^+^ (mmol/L)** | 4.72 ± 0.28 | 4.65 ± 0.20 | 4.57 ± 0.19 | 4.75 ± 0.21 | 4.84 ± 0.25 | 4.66 ± 0.13 |
| **Serum creatinine (mg/[dL.100 g])** | 0.12 ± 0.01 | 0.17 ± 0.02 * | 0.19 ± 0.02 ** | 0.17 ± 0.02 | 0.14 ± 0.01 | 0.15 ± 0.01 |
| **Serum osmolality (mOsm/kg)** | 310.88 ± 2.36 | 304.44 ± 2.63 | 302.18 ± 2.34 * | 316.11 ± 8.08 | 300.78 ± 1.61 ** | 304.50 ± 3.09 |
| **Total bilirubin (mg/dL)** | 8.15 ± 0.21 | 9.45 ± 0.44 * | 7.62 ± 0.91 | 8.61 ± 0.50 | 8.27 ± 0.59 | 8.06 ± 0.57 |
| **AST (IU/L)** | 509.14 ± 76.10 | 615.75 ± 79.07 | 530.00 ± 88.61 | 1022.89 ± 253.93 | 462.11 ± 38.53 | 465.11 ± 26.15 |
| **ALT (IU/L)** | 68.38 ± 6.12 | 76.63 ± 9.57 | 80.00 ± 7.60 | 91.11 ± 10.26 | 72.78 ± 6.69 | 82.67 ± 7.98 |
| **Alkaline phosphatase (IU/L)** | 566.50 ± 62.92 | 438.38 ± 31.36 | 405.73 ± 26.83 * | 376.67 ± 20.85 ** | 423.11 ± 36.53 | 410.89 ± 54.13 |
| **Creatine kinase (IU/L)** | 586.63 ± 72.03 | 640.22 ± 142.49 | 810.36 ± 209.00 | 830.50 ± 182.17 | 473.56 ± 85.25 | 428.00 ± 47.40 |
| **Serum cholesterol (mg/dL)** | 138.88 ± 13.87 | 153.67 ± 22.40 | 141.55 ± 11.35 | 138.56 ± 10.03 | 147.33 ± 15.56 | 130.11 ± 9.58 |
| **Serum albumin (g/dL)** | 2.37 ± 0.11 | 2.25 ± 0.11 | 2.23 ± 0.11 | 2.32 ± 0.06 | 2.26 ± 0.10 | 2.22 ± 0.15 |

Values are expressed as mean ± SEM. n, number of rats; AST, aspartate aminotransferase; ALT, alanine aminotransferase. * p ≤ 0.05, ** p ≤ 0.01, *** p ≤ 0.001 compared with vehicle. † p ≤ 0.05, †† p ≤ 0.01 compared with the equivalent dose of atorvastatin (NCX 6560 17.5 mg/kg/day vs. atorvastatin 15 mg/kg/day and NCX 6560 11.7 mg/kg/day vs. atorvastatin 10 mg/kg/day).

**Supplementary table 2**. Characteristics and biochemical parameters of control and 13-week CCl_4_-induced cirrhotic rats after a 10-day treatment.

|  | **CCl_4_-vehicle** | **CCl_4_-atorvastatin**  **(15 mg/kg/day)** | **CCl_4_-NCX 6560**  **(17.5 mg/kg/day)** | **Control** |
| --- | --- | --- | --- | --- |
| **n** | 8 | 12 | 13 | 8 |
| **Body weight (g)** | 354,45 ± 9,91 | 362,13 ± 8,85 | 344,88 ± 10,79 | 397,05 ± 12,55 * |
| **Weight loss during treatment (g)** | 3,08 ± 1,37 | 10,15 ± 3,67 | 1,59 ± 2,50 | N/A |
| **Urinary volume (mL/h)** | 0,25 ± 0,05 | 0,21 ± 0,05 | 0,30 ± 0,05 | 0,32 ± 0,15 |
| **Serum Na^+^ (mmol/L)** | 143,50 ± 0,59 | 141,89 ± 0,74 | 142,42 ± 0,65 | 141,93 ± 0,63 |
| **Serum K^+^ (mmol/L)** | 5,00 ± 0,18 | 4,98 ± 0,21 | 5,34 ± 0,24 | 5,11 ± 0,20 |
| **Serum creatinine (mg/[dL.100 g])** | 0,25 ± 0,01 | 0,24 ± 0,02 | 0,24 ± 0,01 | 0,24 ± 0,01 |
| **Serum osmolality (mOsm/kg)** | 312,20 ± 1,72 | 311,67 ± 3,96 | 306,62 ± 1,74 | 311,00 ± 4,54 |
| **Total bilirubin (mg/dL)** | 0,08 ± 0,02 | 0,09 ± 0,02 | 0,11 ± 0,02 | 0,09 ± 0,02 |
| **AST (IU/L)** | 261,75 ± 47,94 | 210,50 ± 18,76 | 266,85 ± 34,62 | 147,88 ± 17,90 * |
| **ALT (IU/L)** | 121,50 ± 48,40 | 120,83 ± 14,55 | 134,46 ± 27,64 | 67,00 ± 12,77 |
| **Alkaline phosphatase (IU/L)** | 94,25 ± 9,08 | 106,67 ± 4,84 | 116,15 ± 8,05 | 85,88 ± 6,49 |
| **Creatine kinase (IU/L)** | 1557,00 ± 229,85 | 1943,50 ± 592,66 | 1855,77 ± 328,62 | 1565,13 ± 279,49 |
| **Serum cholesterol (mg/dL)** | 64,20 ± 5,99 | 67,70 ± 3,40 | 74,23 ± 5,05 | 61,88 ± 2,74 |
| **Serum albumin (g/dL)** | 2,70 ± 0,05 | 2,71 ± 0,04 | 2,88 ± 0,07 † | 2,94 ± 0,06 ** |

Values are expressed as mean ± SEM. CCl_4_, cirrhotic rats induced by carbon tetrachloride; n, number of rats; N/A, not applicable; AST, aspartate aminotransferase; ALT, alanine aminotransferase. * p ≤ 0.05, ** p ≤ 0.01 compared with CCl_4_-vehicle. † p ≤ 0.05 compared with CCl_4_-atorvastatin (15 mg/kg/day).
